# More Research Is Necessary to Establish the Ergogenic Effect of Caffeine in Female Athletes

**DOI:** 10.3390/nu11071600

**Published:** 2019-07-15

**Authors:** Juan José Salinero, Beatriz Lara, Ester Jiménez-Ormeño, Blanca Romero-Moraleda, Verónica Giráldez-Costas, Gabriel Baltazar-Martins, Juan Del Coso

**Affiliations:** Exercise Physiology Laboratory, Camilo José Cela University, 28692 Madrid, Spain

Dear Editor-in-Chief,

Today, there is a significant gap in research on the ergogenicity of caffeine, and on sports nutrition in general: the benefits/drawbacks for a given substance are typically assumed for the whole population of athletes when most of the evidence is supported by investigations with only male samples. As a result of this assumption, acute pre-exercise ingestion of 3–9 mg/kg of caffeine is considered an effective strategy to increase sports performance [1], while data on urine caffeine concentration indicates that the use of caffeine in sport is similar in both sexes [2]. A few recent investigations using women as study samples, have also found that caffeine increases sports performance [3,4,5,6]. However, evidence regarding the overall ergogenicity of caffeine in women is much scarcer than in men, and it seems unsafe to conclude that the ergogenic effect of a moderate dose of caffeine is of similar magnitude in men and women.

A search for published studies on the effects of caffeine on physical performance in PubMed and Scopus, following with the Preferred Reporting Items for Systematic Review and Meta-Analyses (PRISMA) guidelines [7], showed a total of 362 original investigations that have compared caffeine to a placebo/control situation, with the measurement of at least one physical performance variable (Figure 1). 

After filters were applied to remove duplicates or publications with unsuitable methodology, the search illustrated that a total of 5321 individuals have been tested to assess caffeine ergogenicity, since the seminal investigation by Costill et al. [8]. From this sample, 703 participants were women, which represents only 13.2% of the total sample. 

Although investigations on this topic have a higher tendency to include women, especially since 2013, women still represent only 16.3% of individuals participating in research carried out in 2018 (Figure 2). In addition, there is no investigation that has measured caffeine ergogenicity in women with doses below 1 mg/kg or above 9 mg/kg, and the number of women in investigations about caffeine effects on speed and muscle power is very low (Table 1). 

Interestingly, there are no investigations measuring the ergogenic effect of caffeine during the different phases of the menstrual cycle, despite the interactions between caffeine and female sex hormones [9]. In fact, it has been found that the effect of caffeine on increasing blood pressure is higher in the follicular than in the luteal phase in female adolescents [10]. All this information indicates that it is still too early to establish that women experience the same ergogenic response to caffeine as men, and further research is needed to describe the optimal conditions of caffeine use in sport and exercise for women. With this Editorial, we want to encourage authors to provide objective information about the dose-effect of caffeine on female athletes’ physical performance. We also want to embolden research focused to determine the magnitude of the ergogenic effect of caffeine during the different phases of the menstrual cycle. The Nutrients’ Special Issue on “Coffee and Caffeine Consumption for Human Health” is open to receive investigations on these topics that hold to “bridge the gap” on the ergogenicity of caffeine in female athletes. 

## Figures and Tables

**Figure 1:**
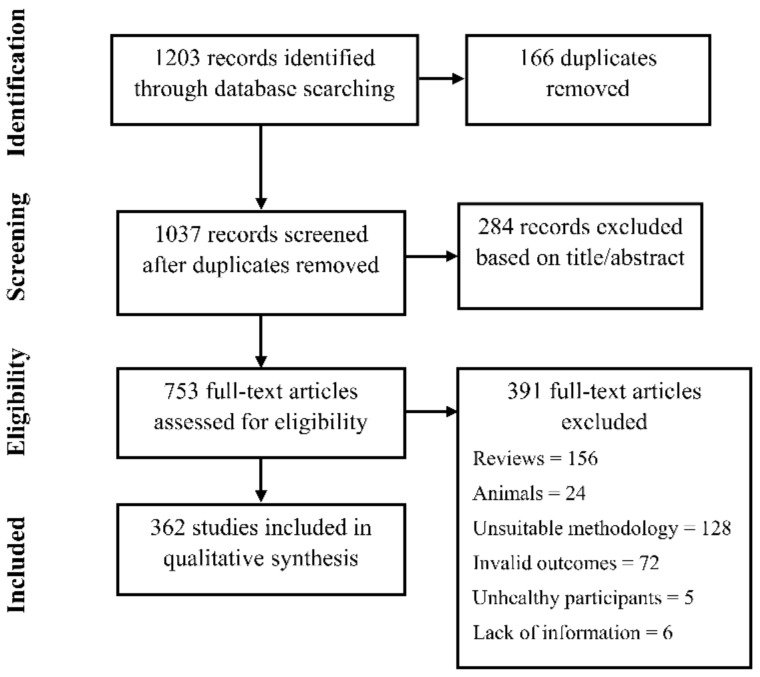
Selection of studies.

**Figure 2:**
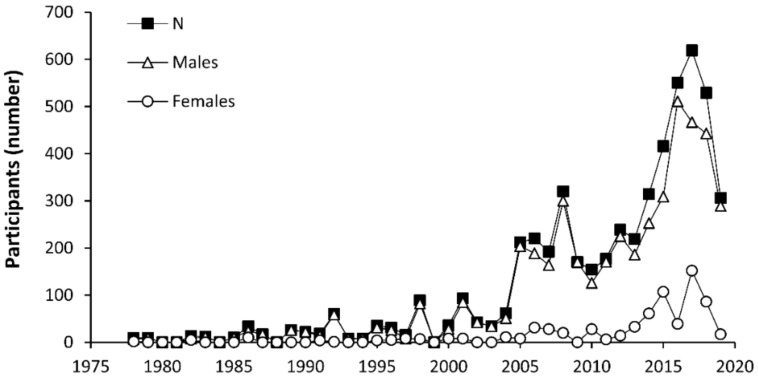
Evolution of the number of participants (*n* = total, males and females) in investigations aimed at determining the ergogenic effects of caffeine.

**Table 1:** Number (frequency) of male and female participants in investigations aimed at determining the ergogenic effects of caffeine depending on dose, type of exercise, and participant’s level.

		Males	Females
Caffeine dose	< 1 mg/kg	10 (100.0%)	0 (0.0%)
	1.0–2.9 mg/kg	608 (90.2%)	66 (9.8%)
	3.0–5.9 mg/kg	2295 (85.2%)	400 (14.8%)
	6.0–9.0 mg/kg	1590 (87.0%)	237 (13.0%)
	>9 mg/kg	115 (100.0%)	0 (0.0%)
Type of exercise	Speed	128 (89.5%)	15 (10.5%)
	Strength	527 (83.1%)	107 (16.9%)
	Power	98 (83.8%)	19 (16.2%)
	Anaerobic-like	587 (88.0%)	80 (12.0%)
	Endurance-like	2019 (89.0%)	249 (11.0%)
	Team-sport	241 (70.9%)	99 (29.1%)
	Other	1018 (88.4%)	134 (11.6%)
Athlete’ level	Trained	2777 (87.8%)	385 (12.2%)
	Active	1421 (85.7%)	237 (14.3%)
	Untrained	420 (83.8%)	81 (16.2%)
